# A Case of Hypermucoviscosity Phenotype of *Klebsiella pneumoniae* Liver Abscess Saved by Damage Control Strategy

**DOI:** 10.1155/2022/6019866

**Published:** 2022-09-29

**Authors:** Keita Sato, Koji Takahashi, Tsukasa Kusuta

**Affiliations:** Department of Surgery, Ise Red Cross Hospital, Japan

## Abstract

Liver abscess caused by the Hypermucoviscosity phenotype of *Klebsiella pneumoniae* (HKp) is characterized by high tissue invasiveness and multiple systemic infections. This leads to sepsis, multiple organ failure, and coagulopathy. An 80 year old man came to our hospital with a complaint of malaise and went into hemorrhagic shock after percutaneous transhepatic drainage of a liver abscess caused by HKp. An emergency laparotomy was performed, but the patient suffered from severe coagulopathy and underwent damage control surgery. HKp liver abscesses must be operated on in the presence of multiple organ failure and disseminated intravascular coagulation (DIC) complications when medical treatment is refractory. In these situations, a two-stage damage control strategy should be considered: hemostasis and infection control at the initial surgery and hepatic resection.

## 1. Introduction

Hypermucoviscosity phenotype of *Klebsiella pneumoniae* (HKp) infection is highly tissue invasive and can easily lead to sepsis, multiple organ damage, and DIC [1]. HKp infection has been reported since the 1980s mainly in East Asia, including Taiwan and South Korea, and rarely in Japan. In this case, we applied a life-saving trauma control strategy (damage control surgery: DCS) to hemostasis and two-stage radical resection of a liver abscess with sepsis and coagulopathy.

## 2. Case Report

**Figure 1:**
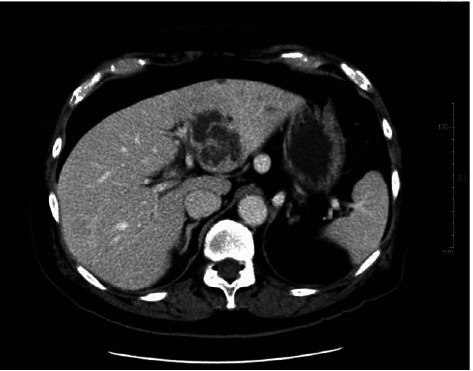
Contrast-enhanced computed tomography on arrival which shows multilocular abscess cavities with a mild enhancement.

The patient was an 80 year old man with a history of prostate cancer (at age 77) and hypertension. He had no history of diabetes mellitus, no oral medication, and only occasional drinking of alcohol. He came to our hospital because of a gradual worsening of weakness that had persisted for several days. On arrival, his consciousness was clear, blood pressure 112/57 mmHg, heart rate 95 beats/min, respiratory rate 20 breaths/min, body temperature 35.8 degrees Celsius, and marked sweating. There was mild tenderness in the upper abdomen. Laboratory examination showed White Blood Cell (WBC) was 8,500/*μ*l and C reactive protein (CRP) was 26.7 ng/dl. CT on arrival (Figure 1) revealed a multifocal lesion (46 mm × 52 mm) in the lateral area of the liver with a contrast effect on the margins. *Klebsiella pneumoniae* was detected in the blood culture collected at the time of admission.

**Figure 2:**
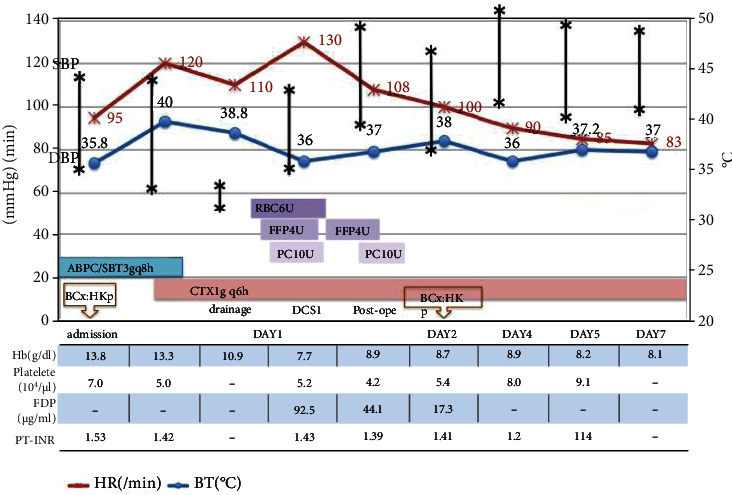
Clinical course after admission. BCx: blood culture, ABPC/SBT: sulbactam/ampicillin, CTX: cefotaxime, NAD: noradrenalin, RBC: red blood cell, FFP: flesh frozen plasma, PC: platelet concentrate, Hb: hemoglobin, and HR: heart rate.

**Figure 3:**
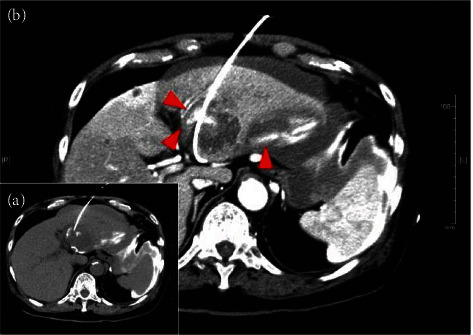
Plain-computed tomography (a) and contrast-enhanced computed tomography (b) after percutaneous transhepatic abscess drainage (PTAD). Extravasation of contrast media and hematoma was observed around the liver and spleen (b, arrowhead).

On the day after admission, his temperature was 40 degrees Celsius, heart rate 120 b.p.m, blood pressure 110/58 mmHg, respiratory rate more than 40 breaths/min, SpO2 88% (10 l/min O_2_ by reservoir face mask), and general condition deteriorated. Blood examination showed a platelet count of 50,000/*μ*l, CRP was 26.7 ng/dl, creatinine was 1.39 mg/dl, T-Bil was 2.9 mg/dl, PT% was 54%, PT-INR was 1.42, and an acute DIC score was 8 (Figure 2). Based on the results of the string test for *Klebsiella pneumoniae*, liver abscess caused by HKp, sepsis, and septic DIC was diagnosed. Abscess drainage was performed to control the infection. A 7.2 Fr pigtail catheter was inserted into the abscess cavity under echocardiography-guided aspiration. Blood was aspirated along with pus. Ultrasonography showed hematoma accumulation around the lateral area of the liver. In addition, a sudden drop in blood pressure and tachycardia was observed. Therefore, intraperitoneal hemorrhage associated with puncture was suspected, and contrast-enhanced CT imaging was urgently performed. When compared with plain CT (Figure 3(a)), a new fluid accumulation and contrast material were seen dorsal to the abscess (Figure 3(b)), suggesting that the abscess had perforated into the abdominal cavity. In addition, extravasation of contrast medium was observed in the perihepatic fluid accumulation (Figure 3(b), arrowhead). The patient was diagnosed to be in shock due to rupture of a liver abscess and intra-abdominal bleeding, and emergency laparotomy was performed (Figure 2).

On laparotomy, a large hematoma was found in the abdominal cavity. A large laceration of about 10 cm was found caudodorsally in the lateral area of the liver (S3). It was thought that bleeding into the abscess cavity and increased internal pressure caused extensive tearing of the liver capsule and bleeding outside the capsule. The hepatic laceration was sutured using absorbable thread. Since the bleeding from the hepatic laceration was persistent and the patient's circulation was unstable, requiring catecholamine even after rapid intraoperative transfusion of 6 units of red blood cells, 4 units of fresh frozen plasma, and 10 units of platelets, we decided to perform damage control surgery (DCS). The liver was packed with gauze from the ventral and dorsal sides of the lateral zone, and the abdomen was simply closed and the patient was returned to the intensive care unit. The operation time was 70 minutes, and the blood loss was more than 2000 ml.

Although the anemia and coagulopathy improved, HKp was detected again in the blood culture collected after the surgery. Therefore, we decided to perform a lateral hepatic resection along with depacking of gauzes.

**Figure 4:**
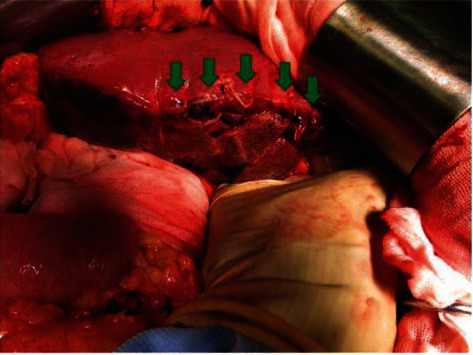
Operative findings. The sutured part (arrow) of lateral hepatic segment was completely free of active bleeding by gauze packing.

The suture closure of the laceration in the lateral area was completely hemostatic with packing (Figure 4, arrow). Lateral hepatectomy was performed under total hepatic blood flow cutoff. There was a very fragile area in the detached section of the liver, and white pus discharge was observed from the same area. The operation time was 4 hours and the amount of blood loss was 500 ml.

**Figure 5:**
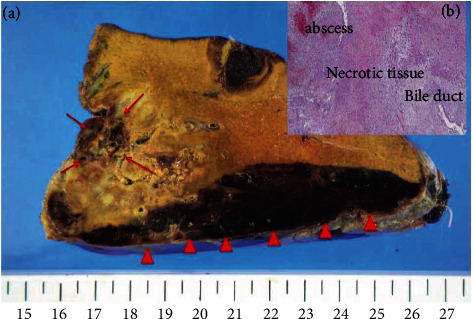
Pathologic findings. Subcapsular hematoma formation of 9.5 × 2.2 × 13 cm (arrowhead) was observed in the lateral area of the liver. Arrows indicate abscess formation of 4.0 × 3.0 × 6.0 cm in the liver lobe.

Following histopathological examination, a 9.5 × 2.2 × 13 cm subcapsular hematoma formation (Figure 5(a), arrowhead) was found on the posterior aspect of the lateral area of the liver. A 4.0 × 3.0 × 6.0 cm geographic abscess formation (Figure 5(a), arrow) was found near the hepatic parenchymal margins. There was an abscess formation near the bile duct (Figure 5(b)), and HKp was also detected in this culture.

His postoperative course was good, and his general condition and coagulopathy improved on the fourth postoperative day. He also had right endophthalmitis by HKp, underwent vitrectomy, and was transferred to another hospital on postoperative day 30.

## 3. Discussion

Antimicrobial agents and percutaneous transhepatic abscess drainage are the mainstay of treatment for bacterial liver abscesses. However, in cases of hyperviscosity, poor drainage, or poor infection control, a surgical approach may be chosen.

In this case, the contrast-enhanced CT findings on admission revealed a multifocal abscess with septa. However, only antimicrobial therapy was administered because it was judged that the drainage would be ineffective due to the small space of each abscess. However, his general condition deteriorated and he developed DIC due to sepsis. During the drainage procedure, the lateral hepatic area ruptured and he went into hemorrhagic shock. At the time of the laparotomy, the patient tended to severe coagulopathy, requiring massive intraoperative blood transfusion and catecholamine administration, so we made the decision to perform DCS.

Although there are some case reports of surgical resection of liver abscesses caused by HKp, it is a high risk to perform liver resection under severe systemic conditions. In Japan, there were 8 case reports of hepatic resection for liver abscess caused by *Klebsiella pneumoniae* with sepsis ([2–8], Table 1). The mean age of the patients was 78.3 years (41-84 years). Diabetes mellitus and heavy alcohol consumption have been pointed out as background disease risks [1]. However, in these 8 cases, there was one case of diabetes mellitus [3], and four cases had no history of diabetes mellitus [4, 6–8]. Four cases [3–6] had multiple foci of infection such as meningitis, vertebral body inflammation, and intraocular inflammation, and 5 cases [4–8] had DIC. The surgical techniques varied widely, ranging from major lobectomy to partial resection, but the prognosis was good with successful surgery, and all patients were discharged alive. Depending on the location of the liver abscess, major lobectomy may be necessary. The degree of invasiveness, including blood loss and operative time, depends on these procedures. There has been no other case in which curative resection was achieved in a two-stage surgery by applying DCS, as in our case. This is a strategy that should be considered as a treatment strategy for very serious cases.

## 4. Conclusion

Surgical approach is considered for liver abscesses caused by hypermucoviscosity type of *K. pneumoniae* when medical therapy is ineffective, but sepsis or DIC may have occurred by this time. The more severe the patient's condition, the shorter and less invasive the hepatic resection should be, but as in this case, the patient may have a coagulopathy or multiple organ failure. In such cases, a two-stage strategy for radical surgery using DCS, which is used in trauma surgery, is necessary.

## Figures and Tables

**Table 1:** Previously reported cases of hepatectomy for liver abscess and severe sepsis caused by hyper mucoviscosity *Klebsiella pneumoniae* in Japan.

Case	Author		Age	Sex	Medical history	Comorbidity	Operation	Outcome	Reference
1	Shiba et al.	2007	84	F	None	Severe sepsis	Lateral segment hepatectomy	Alive	[2]
2	Tomiyama et al.	2007	77	F	DM Hypertension cerebral infarction	Severe sepsis Meningitis	Left hepatectomy	Alive	[3]
3	Morii et al.	2012	69	F	None	Severe sepsis Spondylitis DIC	Partial hepatectomy	Alive	[4]
4	Kittaka et al.	2013	61	M	Alcoholic hepatitis	Severe sepsis Meningitis DIC endophthalmitis	Lateral segment hepatectomy	Alive	[5]
5	Hashimoto and Sumida	2018	69	M	None	Sepsis DIC endophthalmitis	Sub segmentectomy (S8)	Alive	[6]
6	Shibasaki et al.	2019	41	M	None	Sepsis DIC	Right hepatectomy	Alive	[7]
7	Imai et al.	2019	65	F	HBV carrier	Sepsis DIC	Left hepatectomy	Alive	[8]
8	Our case	2021	80	M	None	Sepsis DIC endophthalmitis	**DCS** Left hepatectomy	Alive	Our case

M: male; F: female; DM: diabetes mellitus; DIC: disseminated intravascular coagulation; DCS: damage control surgery.

## Data Availability

The authors do not have additional data or supplementary files.
